# Genomics and 20 years of sampling reveal phenotypic differences between subpopulations of outmigrating Central Valley Chinook salmon

**DOI:** 10.1111/eva.13705

**Published:** 2024-06-03

**Authors:** Tasha Q. Thompson, Shannon O'Leary, Sean O'Rourke, Charlene Tarsa, Melinda R. Baerwald, Pascale Goertler, Mariah H. Meek

**Affiliations:** ^1^ Michigan State University East Lansing Michigan USA; ^2^ Wild Salmon Center Portland Oregon USA; ^3^ Saint Anselm College Manchester New Hampshire USA; ^4^ University of California Davis California USA; ^5^ Cary Institute of Ecosystem Studies Millbrook New York USA; ^6^ California Department of Water Resources Sacramento California USA; ^7^ Delta Stewardship Council Sacramento California USA; ^8^ The Wilderness Society Bozeman Montana USA

**Keywords:** conservation, diversity, genomics, Salmon

## Abstract

Intraspecific diversity plays a critical role in the resilience of Chinook salmon populations. California's Central Valley (CV) historically hosted one of the most diverse population complexes of Chinook salmon in the world. However, anthropogenic factors have dramatically decreased this diversity, with severe consequences for population resilience. Here we use next generation sequencing and an archive of thousands of tissue samples collected across two decades during the juvenile outmigration to evaluate phenotypic diversity between and within populations of CV Chinook salmon. To account for highly heterogeneous sample qualities in the archive dataset, we develop and test an approach for population and subpopulation assignments of CV Chinook salmon that allows inclusion of relatively low‐quality samples while controlling error rates. We find significantly distinct outmigration timing and body size distributions for each population and subpopulation. Within the archive dataset, spring run individuals that assigned to the Mill and Deer Creeks subpopulation exhibited an earlier and broader outmigration distribution as well as larger body sizes than individuals that assigned to the Butte Creek subpopulation. Within the fall run population, individuals that assigned to the late‐fall run subpopulation also exhibited an earlier and broader outmigration distribution and larger body sizes than other fall run fish in our dataset. These results highlight the importance of distinct subpopulations for maintaining remaining diversity in CV Chinook salmon, and demonstrates the power of genomics‐based population assignments to aid the study and management of intraspecific diversity.

## 1 INTRODUCTION

Intraspecific diversity promotes stability and resilience in wild populations (Braun et al., 2016; Carlson & Satterthwaite, 2011; Greene et al., 2009; Hilborn et al., 2003; Schindler et al., 2010). Diversity can manifest in many forms (e.g., phenotypic, spatial, temporal, genetic, etc.) and occur across scales ranging from species down to individuals within small subpopulations. In each case, diversity can provide benefits via the “portfolio effect”, whereby environmental fluctuations that negatively impact one component of diversity may be relatively benign (or even positive) for another (Braun et al., 2016; Doak et al., 1998; Figge, 2004; Moore et al., 2014). This reduces variance in population size over time. Declines in diversity increase vulnerability to sudden and dramatic population size contractions and the risk of extirpation (Carlson & Satterthwaite, 2011; Moore et al., 2010).

Chinook salmon (*Oncorhynchus tshawytscha*) provide a prime example of a species where intraspecific diversity plays a critical role in population resilience (Braun et al., 2016; Carlson & Satterthwaite, 2011; Moore et al., 2010). Chinook salmon are born in freshwater streams across the Pacific Rim, spend several months to more than a year in freshwater, migrate to the ocean as juveniles to spend 1–6 years feeding and growing, then return to their natal river to spawn (Quinn, 2005). Substantial variation can exist at every stage of this lifecycle (e.g., egg incubation time, length of residence in freshwater, juvenile outmigration time, ocean distribution, adult return time, spawn time, etc.). In addition, many populations are composed of distinct subpopulations that exhibit environmental and/or phenotypic diversity both between and within subpopulations, further increasing portfolio complexity (Braun et al., 2016). Greater diversity is associated with greater population stability over time, while Chinook salmon populations with little diversity are susceptible to sudden collapse (Braun et al., 2016; Carlson & Satterthwaite, 2011).

California's Central Valley (CV) historically hosted one of the most diverse population complexes of Chinook salmon in the world (Herbold et al., 2018; Williams, 2006; Yoshiyama et al., 1998). Four distinct runs (i.e., adult spawning migration times)–winter run, spring run, fall run, and late‐fall run–utilized distinct spatial and temporal spawning habitat throughout the Sacramento and San Joaquin River systems. For juvenile Chinook, the rich floodplains, extensive delta, and protected San Francisco Bay provided myriad opportunities for diversity in rearing and migration behaviors (Sturrock et al., 2015; Whipple et al., 2012). Multiple subpopulations of each run existed between separate tributaries (e.g., historically there were at least 18 independent spring‐run populations across the Sacramento and San Joaquin basins) (Lindley et al., 2004). This immense variation allowed Chinook salmon to thrive in a geographic region with extreme variability in year‐to‐year climatic conditions (Dettinger, 2011; Herbold et al., 2018).

Anthropogenic factors have dramatically impacted Chinook salmon diversity in the CV, with severe consequences for population resilience (Carlson & Satterthwaite, 2011; Yoshiyama et al., 1998, 2000). Impassable dams have eliminated all historical winter run spawning habitat, all historical spring run habitat in the San Joaquin system, and most putative historical late‐fall run habitat (Yoshiyama et al., 1998). The winter run is listed as Endangered under the Endangered Species Act (ESA). Individual spring run subpopulations have been reduced from 18 to 4 and the spring run is listed as Threatened under the ESA (US Office of the Federal Register, 1999). The fall run has become homogenized and highly synchronized across tributaries after nearly a century of hatchery practices that have included deliberate transfers of stocks throughout the basin and large‐scale releases of smolts into the San Francisco Bay, leading to high stray rates approaching 100% in some cases (Satterthwaite & Carlson, 2015; Sturrock et al., 2019; Williamson & May, 2005). These massive declines in diversity have resulted in a population complex vulnerable to sudden and severe decreases in census size. Indeed, a weakened portfolio effect has been implicated in fisheries closures and the collapse of CV populations (Carlson & Satterthwaite, 2011).

Maintaining and promoting remaining diversity in the CV is therefore a critical concern for conservation and fisheries management. Currently, the management of diversity is primarily focused on the three evolutionarily significant units (ESUs) that represent the winter run, spring run, and fall run (which also includes the late‐fall run) (Lindley et al., 2004). The phenological and demographic distinctness of these units facilitates both their study and management and the ESA statuses of the winter and spring runs provide conservation mandates. However, major management challenges remain (Nelson et al., 2022). A primary obstacle is the difficulty of distinguishing different runs during the juvenile phase of the life cycle, which has impeded the development of basic monitoring tools such as juvenile production estimates for individual runs (Cordoleani et al., 2020; Nelson et al., 2022). However, new genetic tools are being implemented to improve monitoring and management of the major populations within the CV (Baerwald et al., 2023; Meek et al., 2020; Nelson et al., 2022).

Other components of diversity are also important contributors to stability both across and within the major runs, but are less understood, often impossible to monitor under the current framework, and are not currently a focus of management (Cordoleani et al., 2020, 2021). Two prime, interrelated examples are (1) variation within the juvenile phase of the Chinook life cycle and (2) subpopulation level diversity (Bourret et al., 2016; Brennan et al., 2019; Cordoleani et al., 2021; Herbold et al., 2018; Nelson et al., 2022; Sturrock et al., 2020). Juveniles exhibit variation in their timing and age of outmigration, size, and habitat utilization (Bourret et al., 2016; Cordoleani et al., 2021; Singer et al., 2020; Sturrock et al., 2015; Williams, 2006), and the relative success of a particular juvenile strategy can vary dramatically depending on how it aligns with environmental factors in a given year (Cordoleani et al., 2021; Herbold et al., 2018; Sturrock et al., 2020). Juvenile survival rates strongly affect the number of adults returning in subsequent years (Michel, 2019; Sturrock et al., 2015). Subpopulation diversity is an important stabilizing force for salmon populations (Carlson & Satterthwaite, 2011; Moore et al., 2010; Schindler et al., 2010) and, despite dramatic losses, several subpopulations persist in both the spring‐run and fall‐run ESUs (Carlson & Satterthwaite, 2011; Lindley et al., 2004; Yoshiyama et al., 2000). Subpopulation diversity and juvenile life histories are also intrinsically related, as variation in juvenile characteristics may exist both within and between subpopulations (Bourret et al., 2016; Singer et al., 2020; Yoshiyama et al., 1998). Thus, factoring subpopulation and juvenile diversity information into management could provide important benefits for maintaining and promoting the portfolio of CV Chinook salmon (Cordoleani et al., 2020).

Currently, length‐at‐date (LAD) designations are used to categorize outmigrating juveniles into major populations of origin (winter, spring, and fall/late‐fall runs). However, LAD designations have been shown to have very high error rates and they do not account for subpopulation diversity within the spring run (Brandes et al., 2021; Nelson et al., 2022). The major populations within the CV are genetically distinct, and a lesser degree of genetic differentiation exists between some spring run subpopulations, as well as fall and late‐fall runs (Banks et al., 2014; Clemento et al., 2014; Hedgecock et al., 2001; Meek et al., 2020). Therefore, genetic approaches hold great potential for improving the CV juvenile monitoring framework. However, genetic approaches have so far seen limited applications in understanding juvenile phenotypic diversity in the CV, especially at the subpopulation level (Cordoleani et al., 2020).

Recently, a study indicated the feasibility of assigning individuals to subpopulations within the spring and fall runs using thousands of loci from next generation sequencing (Meek et al., 2020). However, while the study demonstrated the ability to distinguish different subpopulations, it did not address questions related to phenotypic diversity at the subpopulation level, and it also relied on high quality, deeply sequenced samples of known subpopulation origins. No study has utilized subpopulation assignments with genomic data to evaluate phenotypic diversity, nor attempted such assignments in sample sets typical of monitoring or archive collections, where samples are of unknown natal origin and may have highly variable DNA qualities and sequencing outcomes.

Here we use next generation sequencing and an archive of thousands of tissue samples collected across two decades during the juvenile outmigration to accomplish three goals:

1. Develop and validate an approach for assigning samples to populations and subpopulations of origin that is efficacious in sample sets with heterogeneous data qualities.
2. Assign archived juvenile samples collected during their outmigration to populations and subpopulations of origin.
3. Evaluate phenotypic diversity in outmigrating juveniles between and within CV Chinook salmon populations.

## 2 MATERIALS AND METHODS

### 2.1 Population assignment design and validation

#### 2.1.1 Training set development

**FIGURE 1:**
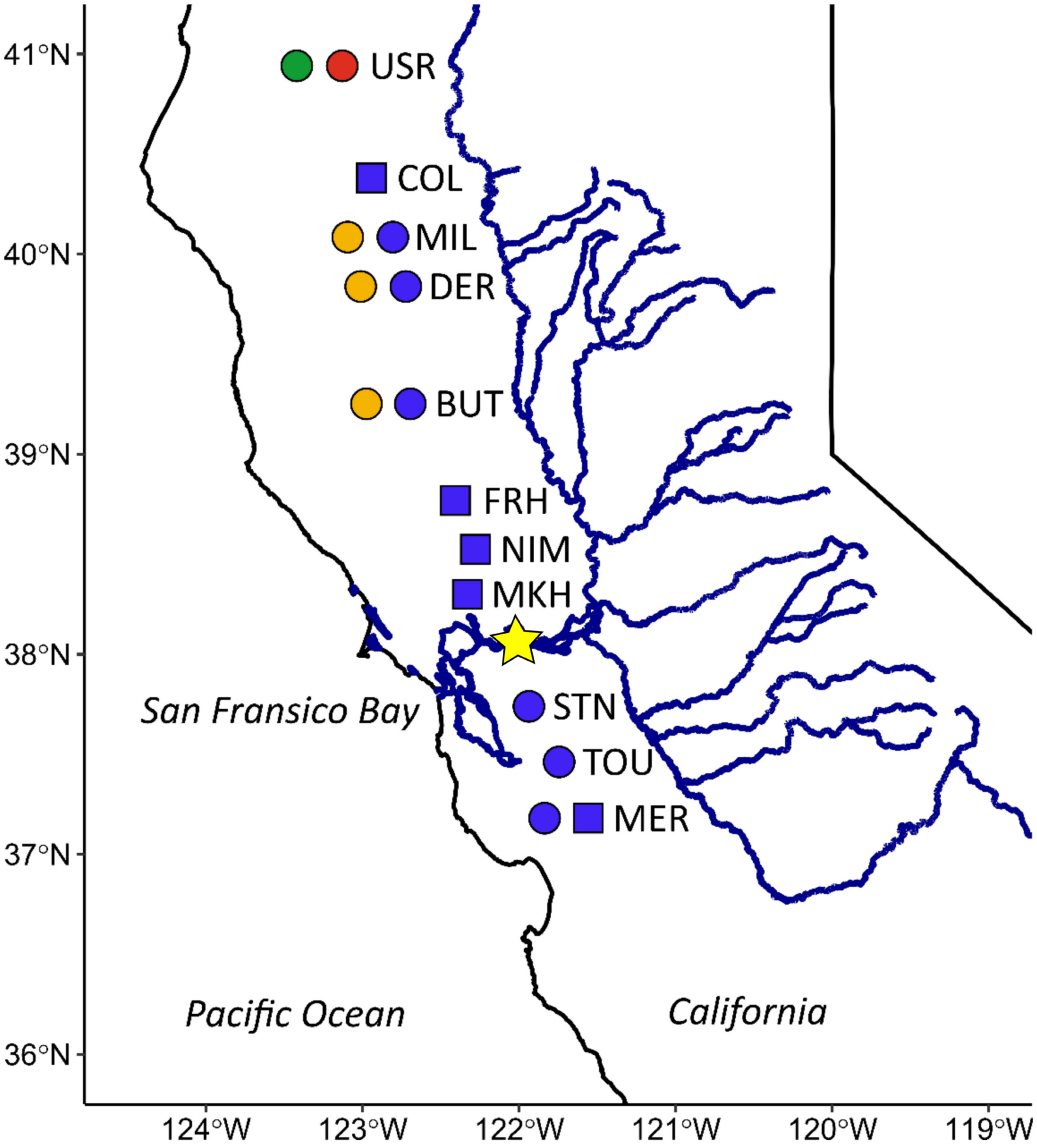
Central Valley Chinook salmon populations present in training sample set. Hatchery populations are represented by squares and natural‐spawning populations by circles. Colors represent the run timing of each population (Spring = orange, Fall = blue, Late‐Fall = red, Winter = green). Abbreviations for tributaries or collection locations: USR (Upper Sacramento River), COL (Coleman Hatchery/Battle Creek), MIL (Mill Creek), DER (Deer Creek), BUT (Butte Creek), FRH (Feather River Hatchery), NIM (Nimbus Hatchery/American River), MKH (Mokelumne River Hatchery), STN (Stanislaus River), TOU (Tuolumne River), MER (Merced River). STN, TOU, and MER are located in the San Joaquin River basin while all other populations are in the Sacramento River basin. The yellow star indicates the location of Chipps Island where juvenile archive samples were collected. Figure is reproduced and modified from O'Leary et al. (2021).

To develop a method for assigning individuals to populations and subpopulations of origin in an archive dataset with highly heterogeneous sample qualities (see below), samples from a previously published and publicly available restriction‐site associated DNA (RAD) dataset of known‐origin CV Chinook salmon (Meek et al., 2020) was utilized to create a training sample set (Table S1). The known‐origin samples included individuals from the three major populations in the CV (winter, spring, and fall/late‐fall) as well as subpopulations within spring and fall/late‐fall. This sample set had previously been used to demonstrate genetic differentiation both across and within major populations (Figure 1; Meek et al., 2020). Hatchery spring run samples were excluded because only wild spring‐run juveniles were expected to be present in the archive data set (see below). The dataset contains only single‐end reads, and these were aligned to the Chinook salmon reference genome (Otsh_V2.0; Christensen et al., 2018) using BWA with the mem algorithm (Li, 2013). Samtools was used to sort the data, index final bam files, and calculate final read counts (Danecek et al., 2021). Samples with greater than 500,000 final alignments were retained for downstream analysis.

A panel of loci was identified from the training set samples using the program ANGSD (Korneliussen et al., 2014). Only reads that mapped uniquely were retained (‐uniqueOnly 1). Quality scores were recalculated around indels using the a BAQ computation (‐baq 1; Li, 2011). Reads with mapping qualities less than 30 and bases with base qualities less than 30 were removed (‐minMapQ 30, ‐minQ 30). Sites were retained if they had a *p*‐value of being variable of 10^−6^ or better (‐SNP_pval 1e‐6). Allele frequencies were calculated from genotype likelihoods using the Samtools model (‐GL 1), a uniform prior (‐doPost 2), and a maximum likelihood approach (‐doMajorMinor 1; Skotte et al., 2012) in order to eliminate sites with an allele frequency less than 0.05 (‐minMaf 0.05). Sites present in less than 50 percent of individuals were removed (‐minInd n). This resulted in a final panel of 9796 single nucelotide polymorphisms (SNPs) that was subsequently used for making population assigments in all datasets described below.

A single‐read sampling approach with ANGSD (‐doIBS 1, ‐doCounts 1) was used to sample one allele from each individual at each locus in the SNP panel. This approach effectively down samples each individual to 1X coverage at each locus, and was utilized in place of genotype calling in order to correct for possible biases due to differences in coverage between the training set and unknown‐origin samples. Preliminary analyses revealed unequal levels of missing data (i.e., “missingness”) between training set samples had strong potential to bias assignments. Therefore, individuals with more than 20% missing allele calls were removed and remaining individuals had their data randomly sampled down to 20% missing data using custom R scripts (see Appendix S1; R Core Team, 2021). The resulting dataset was used as the training set input for DAPC (Jombart et al., 2010; R Core Team, 2021) in all assignment analyses described below.

#### 2.1.2 Assignment approach design

**FIGURE 2:**
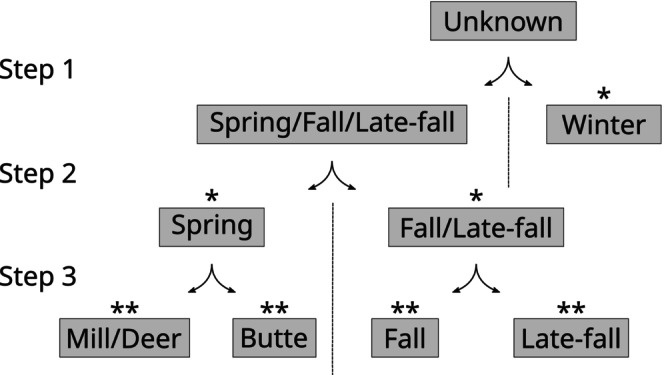
Stepwise assignment approach design. Samples were assigned to populations and subpopulations of origin through a series of steps corresponding to the hierarchy of genetic differentiation within CV Chinook salmon found in Meek et al. (2020). A single asterisk (*) indicates a major population that corresponds to one of the three ESUs within the CV. Double asterisks (**) indicate subpopulations within the major populations. “Spring”, “Mill/Deer”, and “Butte” are wild spring‐run populations, “Late‐fall” are of natural spawning origin, and “Fall” may be of either hatchery or natural origin (see Methods).

Next, we developed a step‐wise approach to assign individuals to populations and subpopulations of origin at progressively finer demographic scales (Figure 2). Samples were drawn from the full training set described above (Table S1) to create targeted training sets for the following step levels that correspond to a hierarchy of demographic differentiation previously identified in the CV (Meek et al., 2020): (1) winter run vs. spring/fall/late‐fall; (2) spring vs. fall/late‐fall; (3a) Mill Creek and Deer Creek spring vs. Butte spring; (3b) fall vs. late‐fall (Figure 2).

While our training set and assignment approach include and account for the most prominent populations and subpopulations within the CV, there a few subpopulations that are not included that warrant explanation. Several spring run subpopulations beyond Mill/Deer and Butte Creeks exist within the CV, the largest of which is the Feather River Hatchery (FRH) spring run. However, FRH spring run genetically assign to the fall run demographic group (likely due to past hatchery introgression between runs) and are 100% adipose‐clipped (marked fish were excluded from our juvenile sample set, see below) (California HSRG, 2012; Meek et al., 2020). Any FRH spring run juveniles that escaped clipping or regenerated their adipose fin would be expected to assign to fall run and constitute only a very small fraction of individuals. The Yuba River natural spawning spring run subpopulation is heavily influenced by, and likely dependent on, straying from the FRH population (Sturrock & Johnson, 2013) and therefore would be expected to also genetically assign to fall‐run. Given the small size of the Yuba River spring run relative to the overall CV fall run (average escapement across years in this study: ~1000 individuals for Yuba River spring run vs. >100,000 individuals for total fall run), such individuals would also constitute only a very small fraction of fall run assigned juvenile samples (Azat, 2023; Nelson et al., 2022). Of the spring run subpopulations outside of the FRH and Yuba River, Butte Creek and Mill/Deer subpopulations together constituted approximately 94% of the estimated average spring run escapement over the years of juvenile samples included in this study (73% Butte Creek and 21% Mill/Deer) (Azat, 2023). The only other spring run subpopulation that may be independent is Battle Creek, which averaged ~3% of estimated total spring run escapement (excluding FRH and Yuba) over the years sampled. Battle Creek spring run samples were not available for inclusion in our training set, and it is currently unclear what population or subpopulation they would assign to (they were previously extirpated for several decades and only began to reestablish in the 1990s; Williams et al., 2016). However, their small population size indicates they are expected to make up only a small proportion of spring run juvenile samples in this study.

Preliminary analyses revealed the importance of balancing the number of training samples in each group at each step. Therefore, at each step, the population with the greater number of individuals in the full training set (Table S1) had samples randomly removed from the training set input data described above so that each population ended up with an equal number of individuals.

Discriminate analysis of principal components (DAPC) in the Adegenet package in R was used to assign individuals to a group at each step (Jombart et al., 2010; R Core Team, 2021). To evaluate error rates and develop assignment thresholds, we conducted a leave‐one‐out analysis at each assignment step. For a given assignment step, one individual from that step's training set was selected for testing and removed from the training set. One individual from the alternate population was also randomly removed in order to keep the number of training samples in each population equal. DAPC was run on the remaining individuals using their known populations of origin (Jombart et al., 2010). The test individual was then treated as an unknown and assigned to a population using the dapc.predict() function. DAPC returned population assignment posterior probabilities, and an assignment call was made for the test individual based on the greatest assignment posterior. Both the call and the posteriors were recorded. To evaluate the relationship between the amount of missing data (i.e., “missingness”) in a sample and assignment accuracy, we repeated the leave‐one‐out analyses by subsampling data from the test individual to progressively greater levels of missingness (ranging from 20% to 99%; the missingness of the training set samples remained unchanged). The SNP panel consisted of 9796 loci, so 20%–99% missingness corresponded to between 7837 and 98 loci with data present in the tested sample. This was repeated for all samples in each step's training set. The results were used to understand how assignment error rates varied across levels of missingness and assignment posterior probabilities, and then facilitate selection of reasonable thresholds for missingness and assignment posteriors. Thresholds were selected with the aim of including as many samples as possible while maintaining low expected assignment error rates, and were specific to each assignment step (see Results; Table S2; Figure S2).

DAPC requires the number of retained principal components (PCs) to be specified, and the choice can influence results (e.g., including too many PCs can lead to over‐fitting of data) (Jombart & Collins, 2015). In order to choose an appropriate number of PCs to include, the leave‐one‐out analyses were repeated using a range of principal components (1–12). This revealed that including between three and five principal components resulted in the lowest error rates, with no clear distinction between choices in that range. Thus, we chose three principal components to use for all downstream DAPC analyses.

#### 2.1.3 Validation of the assignment approach

In order to validate this approach, we applied our assignment method to an independent publicly available data set of known origin samples generated and described in Baerwald et al. (2023) (Table S1). The validation data had been generated from paired‐end whole genome sequencing data and contained widely variable levels of coverage. Thus, it provided an opportunity to test the robustness of our assignment approach in a distinct dataset of variable sample qualities.

Fastq files for each individual in the validation set had sequencing adapters removed with Cutadapt (Martin, 2011) and were aligned and processed using the same methods as the training set, except duplicates were removed using Samtools ‐markdup and the data set was filtered to only retain properly‐paired reads (Danecek et al., 2021; Li, 2013). Single read sampling in ANGSD (Korneliussen et al., 2014) was conducted at the sites in the SNP panel identified with the training set (see above) Thirty out of the 9796 sites in the panel were missing data in all validation samples, but otherwise the full SNP panel was utilized.

The single read sampling step produces a file that contains 0 and 1 coding for major and minor alleles, respectively. Because single read sampling is performed separately for the training set and validation dataset, inconsistent coding between the validation file and the training set file would arise if a given allele is the major allele in one dataset but the minor allele in the other. To account for this, we compared major and minor alleles at each SNP in each dataset and switched the coding in the validation file where necessary to be consistent with the training set file (see Supplemental Materials). The corrected data was used as input for DAPC (Jombart et al., 2010; R Core Team, 2021).

Samples were assigned to populations using the stepwise approach described above (Figure 2). For a given step, DAPC was run on the balanced training set for that step using three PCs (Jombart et al., 2010; R Core Team, 2021). Next, the dapc.predict() function was used to assign the validation samples to one of that step's populations. An assignment call at each step was made based on the largest population posterior, and both the call and posteriors were recorded. The missingness and posterior thresholds selected based on the leave‐one‐out analyses (see Table S2) were applied to identify low‐confidence assignments. The calls were then compared to the known origins of the samples to evaluate error.

### 2.2 Assignment of archived juvenile samples

#### 2.2.1 Sample acquisition, sequencing, and initial processing

To generate a sample set for evaluating subpopulation level differences in juvenile outmigration characteristics in CV Chinook salmon, we obtained samples from the California Department of Fish and Wildlife (CDFW) tissue archive. The samples had been collected across more than 20 years (1996–2018) during midwater trawling at a monitoring station (Chipps Island) in the CV delta below the confluence of the Sacramento and San Joaquin Rivers (Figure 1). Since 1994, year‐round sampling has been conducted at Chipps Island (Brandes & McLain, 2001). In general, ten 20‐min trawls were conducted 3–7 days per week within a 3 km section of river near Chipps Island. Trawls are conducted in three channel locations (north, south, and middle), and both flow directions (Brandes & McLain, 2001; Pyper et al., 2013). Sampling effort had a degree of variability within and across the 20 years of samples included in this study. For example, the number of trawling days per week was comparatively high during experimental releases of coded‐wire‐tagged juveniles (typically April–May and December–January), and trawling was suspended for about a month in 2007 and several months in 2008 due to high incidental take of delta smelt (Brandes & McLain, 2001; Pyper et al., 2013). This variability did not preclude testing for phenotypic differences between groups of samples assigning to different populations (i.e., because all samples within the archive were subject to the same sampling effort variability). However, it influenced our ability to define comprehensive phenotypic distributions (e.g., outmigration timing distributions) for individual groups (see below).

A total of 5287 samples were obtained from the archive (Table S1). Samples were selected across all dates and size ranges in the archive to closely reflect the diversity present in the complete collection (Figure S1). Only samples from fish with intact adipose fins were included. This was expected to exclude all or nearly all fish from hatchery programs with 100% clipping targets (all winter run, spring run, and late‐fall run hatchery programs). However, only 25% of CV hatchery fall run fish are adipose clipped (California Hatchery Scientific Review Group (California HSRG), 2012). This sample set is therefore expected to contain a large number of unclipped hatchery fall run fish. Extensive straying and very high numbers of hatchery fish among natural spawners has led to near‐complete homogenization of the CV fall run regardless of origin (Sturrock et al., 2019; Williamson & May, 2005), and it is not possible to genetically distinguish natural spawners from hatchery fall run fish. The low marking rate of hatchery fall run also means it is generally not possible to distinguish the majority of hatchery origin from non‐hatchery origin fall run through other means, and the high straying rate of fall run hatchery fish results in a substantial number of unmarked hatchery fish on natural spawning grounds. Here, assessments of fall run outmigration characteristics will not distinguish between natural and hatchery origin juveniles.

DNA was extracted from the samples using a SPRI bead‐based protocol, and restriction site‐associated DNA (RAD) sequencing was performed on the extracted DNA (Ali et al., 2016). SbfI was used as the restriction enzyme. Paired‐end 150 base‐pair RAD libraries were sequenced on an Illumina NovaSeq 6000 S4 flowcell. The program deML (Renaud et al., 2015) was used to split raw sequencing files using plate barcodes. Custom scripts requiring a perfect barcode match were used to split RAD plate data into files for individual samples. Fastq files underwent the same alignment and processing steps as the training set files, except that samtools was also used to remove duplicates and reads that were not properly paired (Danecek et al., 2021; Li, 2013). Initial DNA quantities and qualities were highly variable, and in some cases low‐quality libraries underwent an additional round of sequencing and processing. In these cases, individual bam files from both sequencing runs were merged together using Samtools merge (Danecek et al., 2021). Samples were included in downstream analyses if they had at least 1000 final aligned reads. Single read sampling in ANGSD (Korneliussen et al., 2014) was conducted on these samples for the panel of 9796 SNPs identified in the training set. None of the SNPs were missing data in all of the juvenile samples, so single‐read sampling data at all 9796 SNPs was included as input for DAPC (see below). As with the validation data set, major and minor alleles in the juvenile single read sampling data were compared to the training set file, and changes to the 0/1 coding were made when necessary to ensure consistent coding between files (see Appendix S1).

#### 2.2.2 Assignment to populations and subpopulations of origin

Samples were assigned to populations using the stepwise approach described above (Figure 2). For a given step, DAPC was run on the balanced training set for that step using three PCs (Jombart et al., 2010). The dapc.predict() function was used to assign the validation samples to one of that step's groups. An assignment call at each step was made based on the largest population posterior, and both the call and posteriors were recorded. The missingness and posterior thresholds identified in the leave‐one‐out analyses (Table S2) were applied to remove low‐confidence assignments.

#### 2.2.3 Calculation of allele frequencies for a marker associated with run timing

Previous work identified a genetic locus (GREB1L) associated with adult migration timing in Chinook salmon (Prince et al., 2017), including in CV populations (Meek et al., 2020; Thompson et al., 2020). To evaluate whether there was an allele frequency shift at this locus between the archived juveniles assigned to different populations, we called genotypes at a SNP located within the GREB1L region (NC_056456.1:13427410; Prince et al., 2017). This SNP is commonly sequenced in RAD‐seq datasets and is thus expedient to use. However, it has been shown to have a relatively high false‐positive rate for the early‐run associated allele compared to more recently developed markers for the GREB1L locus that are not on RAD‐tags (Thompson et al., 2018, 2020). While this SNP is therefore not perfectly diagnostic for run timing genetic variation, previous work showed a strong allele frequency shift at this SNP between the early run CV populations (winter and spring run) and the fall/late‐fall run population (Meek et al., 2020). A similar shift should therefore be observed in our archive juvenile samples assigned to different populations, and would support the efficacy of the juvenile assignments for downstream population comparisons.

The program ANGSD (Korneliussen et al., 2014) was used to call genotypes at the GREB1L SNP. The standard filtering described above was combined with genotype calling (‐doGeno 4) for the individual SNP (−r NC_056456.1:13427410). In addition, for each sample, the posterior probability of the genotype call was calculated (‐doGeno 16). Genotypes were subsequently filtered to include only calls with posteriors >0.8. Allele frequencies at the SNP were then calculated for juveniles assigned to each major CV population.

### 2.3 Comparison of outmigration characteristics between and within populations

To test for differences in outmigration timing between population and subpopulation groups, we calculated the median outmigration date of each assignment group. Medians for each group within an assignment step were compared, and significant differences were detected by permutation testing using the pairwisePercentileTest() function in the R package rcompanion (Mangiafico, 2015; R Core Team, 2021). Ten thousand repetitions were used to generate a *p*‐value for the observed empirical difference in medians between groups.

Differences in fork lengths between subpopulations were also evaluated. Median fork lengths for each group were calculated, and the significance of the difference between groups was evaluated using 10,000 permutations as described above for outmigration timing.

The application of posterior assignment thresholds results in the removal of individuals with less confident assignments and is intended to reduce assignment errors. However, it also likely removes some correctly assigned individuals. It is unknown whether the removal of correctly assigned fish that appear to exhibit relatively low differentiation (e.g., perhaps due to recent ancestry from strays) could affect results. To explore how our posterior assignment thresholds were influencing our results, we recalculated medians and performed permutation testing to compare outmigration dates and fork lengths (as described above) without excluding any samples based on posterior assignment thresholds (missingness thresholds remained in place). While this is expected to somewhat increase the *p*‐values of comparisons due to increased assignment error rates, severe declines in significance may indicate bias from the exclusion of accurately assigned samples with relatively low differentiation.

Finally, we visually compared the outmigration timing and fork length distributions of each population and subpopulation using violin and boxplots, both as aggregates and in individual years. Variability in sampling at Chipps Island (see above) likely influenced the distribution of our overall dataset (e.g., some time periods when fish were likely present at Chipps Island are missing from our dataset because trawling was suspended (Brandes & McLain, 2001; Pyper et al., 2013). Thus, while these visualizations capture substantial portions of the distributions of each population and subpopulation and are useful for identifying patterns and differences between populations, they are not meant to comprehensively define the distributions for any group. This is especially true for individual years, where sample sizes are often small.

## 3 RESULTS

### 3.1 Leave‐one‐out and validation results

**FIGURE 3:**
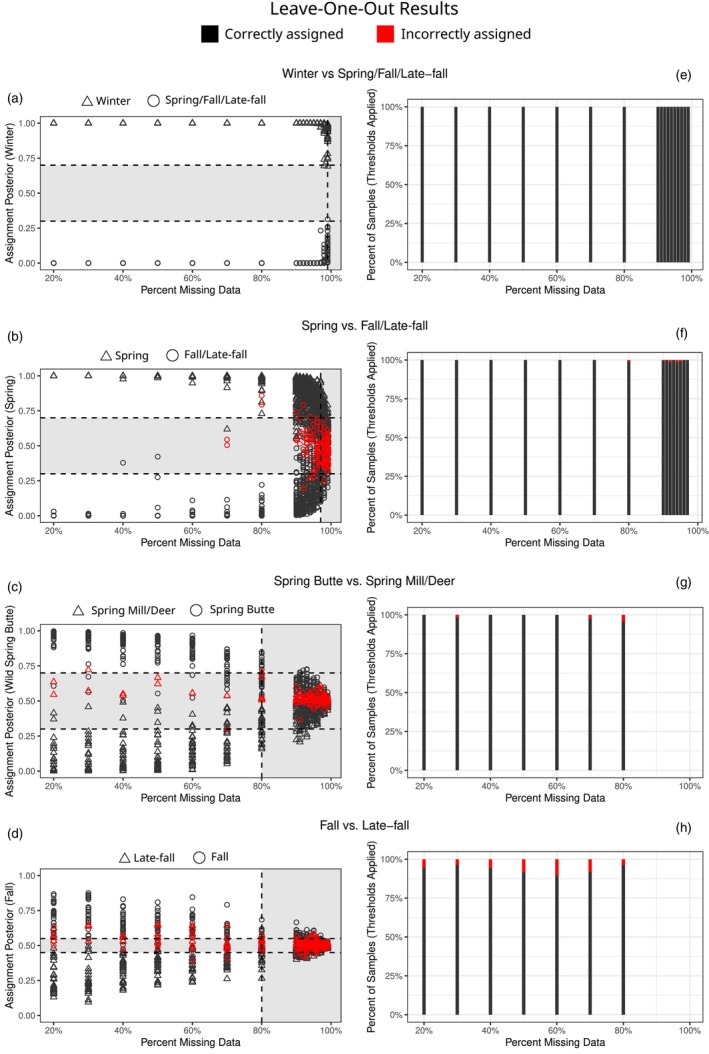
Leave‐one‐out analysis results. (a–d) Each point represents the result for one test sample at a given level of missing data. Shape indicates the true population of the sample. Color indicates whether the sample was assigned to the correct population (black = correct assignment, red = incorrect assignment). The dashed lines and gray‐shaded regions indicate the missingness (vertical lines) and posterior assignment thresholds (horizontal lines) chosen for each step to minimize erroneous assignments (i.e., samples falling within the gray regions had relatively high error rates and thus were considered to have low‐confidence assignments and would be excluded from further analysis). (e–h) Stacked bar graphs indicating the proportions of samples correctly (black) and incorrectly (red) assigned at each level of missingness after all assignment thresholds were applied (missingness levels exceeding the threshold of a given step are not shown because all samples above those levels are excluded). See Figure S2 for a comparison of assignment rates before and after threshold applications.

The leave‐one‐out analyses revealed that the degree to which missingness affected error rates varied for the different assignment steps (Figure 3; Figure S2). For winter vs. spring/fall/late‐fall, no errors in assignment appeared for up to 99% missing data in the test sample. A very high tolerance for missingness at this step was unsurprising given the strong differentiation between winter run and all other CV Chinook populations (e.g., Meek et al., 2020 calculated Fst between winter run and all other CV populations to be ~0.15). For spring vs. fall assignments, error rates ranged from zero for all missingnesses below 60% up to 27% error at 99% missingness (Figure 3, Figure S2). For assignment to spring subpopulations, assignment error rates were 3% for a missingness of 20%, and exceeded 20% error rates for some missingnesses greater than 90% (Figure 3, Figure S2). At each of these steps, most incorrectly called individuals had relatively low posterior probabilities associated with their population assignments. We therefore defined assignment calling thresholds based on missingness and posterior probabilities for each step that were expected to maximizing the number of samples included while also maintaining very low assignment error rates (Table S2; Figure 3; Figure S2). All samples in the validation dataset assigned correctly under these thresholds (Figure S3).

Fall vs. late‐fall assignments had relatively high error rates compared to the above analyses, with error rates near or above 20% for levels of missingness exceeding 60%, and error rates near 10% for lower missingnesses (Figure 3, Figure S2). Posterior assignment probabilities were also low even for correctly assigned samples relative to the other steps. These results are not surprising given the low genetic differentiation between fall and late‐fall compared to all other populations and subpopulations tested here (Meek et al., 2020). To limit error rates while still allowing a sufficient number of passing samples for analyses (see below), we set a missingness threshold of 80% and required an assignment posterior >0.55 (Table S2; Figure 3; Figure S2). Late‐fall samples were somewhat more likely to be erroneously called as fall than the reverse, and these thresholds eliminated almost all false‐positive late‐fall calls (Figure S4). The validation dataset further supported the efficacy of this approach, as no erroneous calls were made in the validation set under these thresholds, and 5 out of 6 late‐fall validation samples would have been assigned correctly even with no thresholds in place (Figure S3). We conclude that our assignment approach and thresholds are highly efficacious and robust to heterogenous data qualities.

### 3.2 Juvenile sample set characteristics

After extraction, DNA quantities and qualities were highly variable, as were read counts and amounts of missing data after sequencing and alignment to the Chinook salmon reference genome (Figure S5). A substantial number of samples produced low quality data. Additional library preparation and/or sequencing on a subset of samples produced only minor improvements in missingness, indicating initial sample quality was likely the main driver of low data quality. This was not unexpected given the broad age range of the samples and likely heterogeneity in initial collection and storage conditions, and represents a common challenge when working with archived samples or field‐collected datasets. In total, 4632 out of 5287 samples had the 1000 reads required for inclusion in downstream analyses.

### 3.3 Major populations exhibit distinct juvenile outmigration timing

Out of 4632 individuals analyzed, 3537 individuals passed missingness and posterior assignment thresholds for the first step (winter vs. spring/fall/late‐fall). Of the individuals that passed, 256 were assigned to winter while 3281 assigned to spring/fall/late‐fall (Table 1; Figure S5). In the next assignment step (spring vs. fall/late‐fall), 2244 individuals passed the thresholds, and 247 assigned to spring and 1997 assigned to fall (Table 1; Figure S5).

**TABLE 1:** Juvenile sample assignment results.

Assignment step	Population or sub‐population	Number of passing thresholds
Step 1	Winter	256
	Spring/Fall/Late‐Fall	3281
Step 2	Spring	247
	Fall/Late‐Fall	1997
Step 3a	Spring Butte	60
	Spring Mill/Deer	26
Step 3b	Fall	1103
	Late‐Fall	31

The frequencies of alleles at the SNP associated with run timing exhibited a very strong shift between individuals assigned to early vs late run populations (early‐run allele frequency among individuals assigned to winter = 0.94; spring = 0.99; fall/late‐fall = 0.19) and were similar to frequencies previously calculated in known‐origin CV samples (Meek et al., 2020). We conclude these results are consistent with expectations (see Methods) and provide support for the efficacy of the juvenile assignments.

**FIGURE 4:**
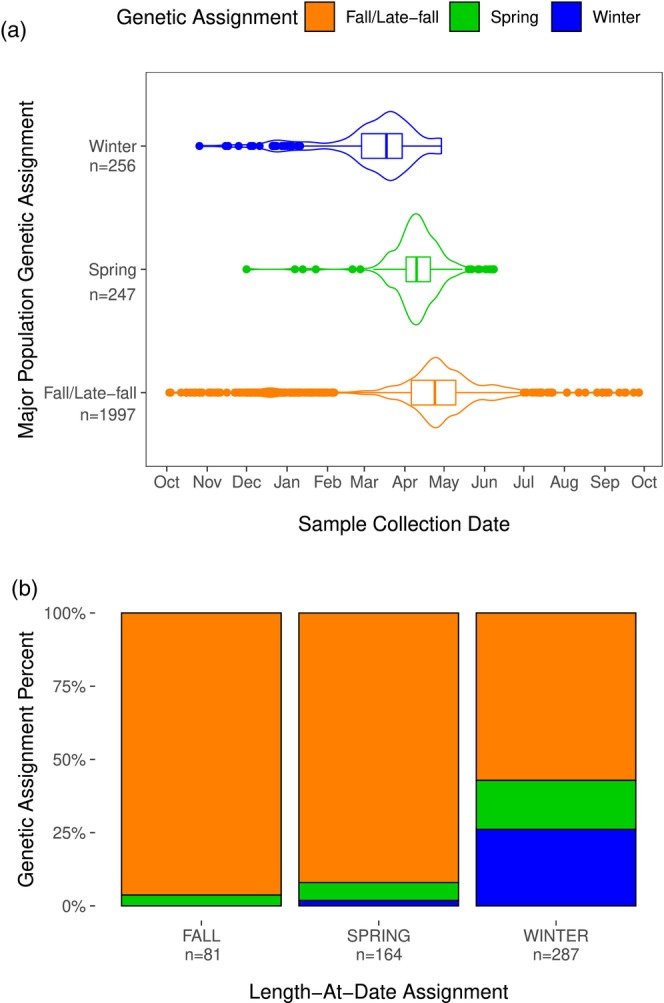
Major Population Genetic Assignments. (a) Violin and boxplots of juvenile outmigration timing (i.e., sample collection date at Chipps Island) of individuals assigned to one of the three major CV Chinook salmon populations. (b) Comparison of major population genetic assignments to LAD population assignments (limited to individuals that had received a LAD assignment from CDFW at the time of collection).

A comparison of the outmigration timing of each major population revealed significantly different median outmigration dates for all groups (*p* < 0.001 for all comparisons; Figure 4a). To ensure the difference in missingness thresholds between steps 1 and 2 hadn't biased the winter‐run comparisons, we also performed this analysis using the same missingsness thresholds for both assignment steps 1 and 2. This resulted in 45 fewer assigned winter‐run (i.e., they were eliminated from analyses because they failed the new missingness threshold), but no difference in overall results. When posterior assignment thresholds were removed, *p*‐values remained <0.001 for all comparisons. We conclude each major population exhibited distinct outmigration timing within our dataset.

Some individuals within the archived juvenile samples had been given major population designation by CDFW based on LAD criteria, which is the currently used framework for monitoring major populations during the juvenile outmigration (Nelson et al., 2022). This framework is especially important for monitoring the two ESUs listed under the ESA (winter run and spring run). To explore the efficacy of the LAD monitoring framework, we compared our genetic population assignments to individuals with LAD designations. Of fish that had received a winter run LAD designation, 26% genetically assigned to winter, 17% assigned to spring, and 57% assigned to fall/late‐fall (Figure 4b). Of fish that had received a spring run LAD designation, only 6% received a genetic assignment to spring run and 92% assigned to fall/late‐fall (Figure 4b). Of fish that had received a fall LAD designation, 96% genetically assigned to fall/late‐fall and 4% assigned to spring (Figure 4b). While our study was not designed to comprehensively quantify errors in LAD designations, these results are consistent with other research that has found very high error rates in LAD estimates and a strong bias towards over‐estimating winter and spring run juveniles (Brandes et al., 2021). We conclude population assignments based on genetics rather than LAD designations could substantially improve the juvenile monitoring framework.

### 3.4 Spring run subpopulations exhibit distinct juvenile outmigration distributions

Out of 247 fish successfully assigned to the major spring population in step 2, 86 passed missingness and posterior assignment thresholds for subpopulation assignment. Of these, 26 assigned to Mill/Deer and 60 assigned to Butte (Table 1, Figure S5).

**FIGURE 5:**
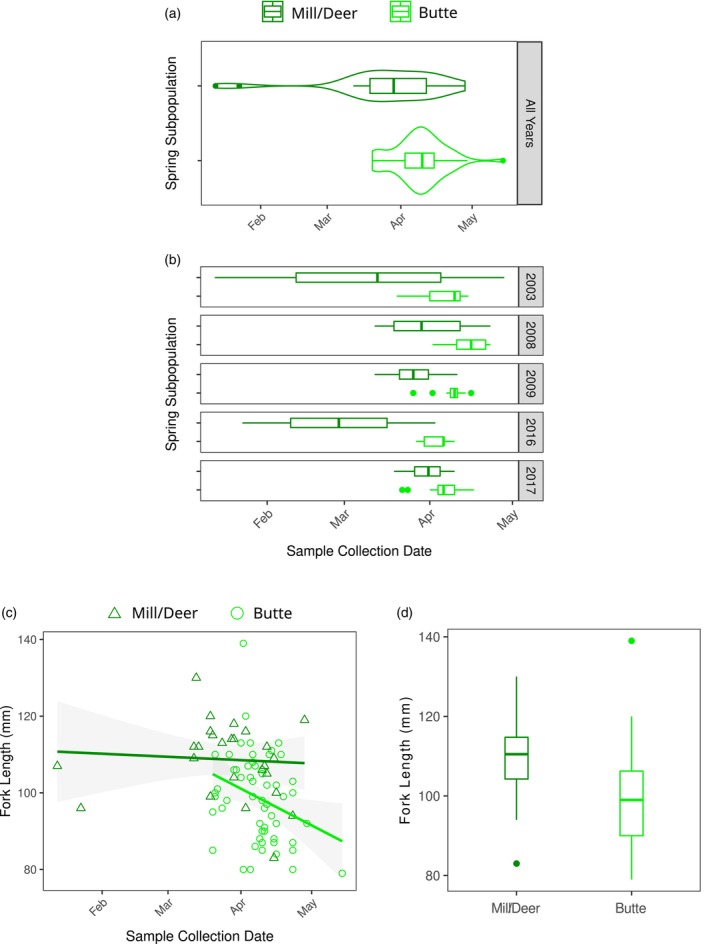
Spring‐run subpopulation juvenile outmigration characteristics. (a) Violin and boxplots of the juvenile outmigration time (i.e., collection time at Chipps Island) for all juveniles successfully assigned to one of the spring‐run subpopulations. (b) Boxplots of juvenile outmigration times for each subpopulation in individual years. Years with fewer than 5 successfully‐assigned samples were excluded (number of samples in included years ranged from 7 to 17). (c) Scatterplot of the outmigration times and fork lengths of fish assigned to a spring‐run subpopulation. Shapes and colors indicate the subpouplation. Points represent individual samples, the lines are trend lines for each subpopulation fit under a linear model. The shadow around the line represents the 95% confidence interval. (d) Boxplot of fork lengths for each spring subpopulation.

A comparison of the outmigration timing of each spring subpopulation revealed significantly different median outmigration dates for Mill/Deer and Butte juveniles (*p* < 0.002; Figure 5a). Mill/Deer samples had a relatively early and broader distribution within our sample set, while Butte samples had a relatively late and compressed distribution. While the numbers of samples in individual years were small, this pattern was nevertheless remarkably consistent across years (Figure 5b). We conclude that Mill/Deer and Butte Creek spring run juveniles exhibit distinct outmigration timing distributions.

We also compared body sizes at outmigration (i.e., fork lengths) between spring run juveniles from Mill/Deer and Butte Creeks. Mill/Deer juveniles had a larger median fork length than Butte juveniles (110.5 vs. 99 mm; *p* = 0.001; Figure 5c,d). For both subpopulations, earlier fish tended to be larger, but Mill/Deer juveniles were consistently larger than Butte at a given date. Recent work using otolith microchemistry has identified age and size diversity of outmigrants to be critical portfolio components in Mill, Deer, and Butte spring run populations (Cordoleani et al., 2024 ). We conclude spring Mill/Deer and Butte Creek juveniles exhibit phenotypic distinctiveness for multiple important traits.

Removing posterior assignment thresholds had negligible effects on results. In the absence of posterior assignment thresholds, the *p*‐value for comparison of median outmigration day was <0.01, and for the comparison of fork length the *p*‐value equaled 0.01. Thus, our posterior assignment thresholds do not appear to be a significant source of bias, and our results appear robust to the increase in error rates expected with the removal of posterior assignment thresholds.

### 3.5 Fall‐run subpopulation assignment and diversity

Out of 1997 fish successfully assigned to the major fall/late‐fall population in step 2, 1134 passed missingness and posterior assignment thresholds for subpopulation assignment. Of these, 31 assigned to late‐fall and 1103 assigned to fall (Table 1, Figure S5).

**FIGURE 6:**
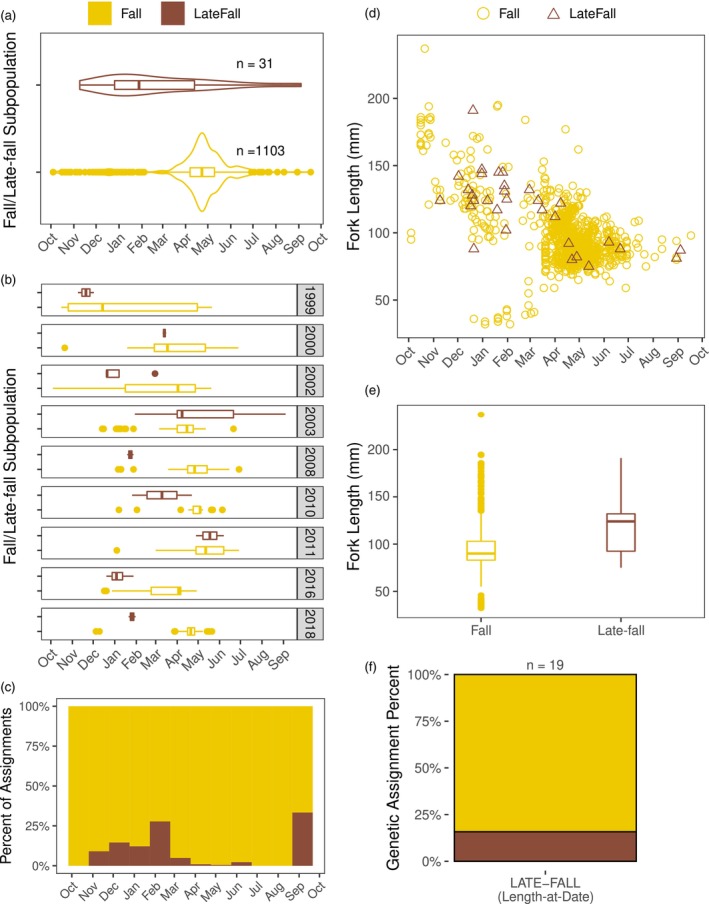
Fall‐ and late‐fall‐run subpopulation juvenile outmigration characteristics. “Fall” and “Late‐fall” refers to genetic population assignments unless noted otherwise. (a) Violin and boxplots of the juvenile outmigration time (i.e., collection date at Chipps Island) for all juveniles successfully assigned to either the fall or late‐fall subpopulations. (b) Boxplot of juvenile outmigration times for each subpopulation in individual years. Years with fewer than 2 late‐fall samples or fewer than 10 fall samples were excluded (the number of samples in included years ranged from 2–5 for late‐fall and 27–233 for fall). Note, sampling timing and effort have varied between years (see Methods). (c) Histogram showing proportion of each subpopulation observed in 30‐day windows across the outmigration period. (d) Scatterplot of the outmigration times and fork lengths of fish assigned to a fall or late‐fall subpopulations. Shapes and colors indicate the subpopulation. Points represent individual samples. (e) Boxplot of fork lengths for each subpopulation. (f) Comparison of genetic assignments to LAD late‐fall‐run designations made by CDFW at time of sample collection.

Next, we compared the outmigration timing distributions of fall and late‐fall assigned fish. The median outmigration times for each subpopulation were highly significantly different (*p* < 0.001 both with and without posterior assignment thresholds in place; Figure 6a), with the median outmigration date for late‐fall occurring more than 2 months before that of fall juveniles. Late‐fall had a strikingly earlier and broader distribution than fall. The earlier timing of late‐fall assigned fish was quite consistent across individual years, although the number of late fall samples in each year was very small (Figure 6b). Given that the number of assigned late‐fall samples was several orders of magnitude less than the number of assigned fall samples, late‐fall individuals made up a highly outsized proportion of all fish sampled between November and March (Figure 6c). The large discrepancies between fall and late‐fall juveniles are particularly notable because assignment error rates are expected to be higher for fall vs. late‐fall compared to other assignment steps (see Figure 3), and errors would be expected to make the timing of each group appear more similar to each other. Therefore, the true difference in median outmigration date between fall and late‐fall may actually be even greater than observed in our data, and the overlap between the runs may be less substantial. We conclude fall and late‐fall juveniles exhibit strongly distinct outmigration timing distributions.

We also compared the fork lengths of fall and late‐fall juveniles. Late‐fall juveniles had a larger median fork length than falls, and this difference was highly significant (124 vs. 90 mm; *p* < 0.001 both with and without posterior assignment thresholds in place; Figure 6d,e). However, fall‐assigned fish exhibited a greater amount of diversity in the sizes of outmigrants than any other assignment group (Figure 6d). Clusters of both the largest and smallest fish in our full dataset assigned almost exclusively to the fall subpopulation. Thus, while a large majority of fall run fish exhibited intermediate sizes, the fall run nevertheless harbors substantial phenotypic diversity in outmigration characteristics, even if that diversity is only present at low frequencies.

We also compared LAD designations for late‐fall run to genetic assignments. Of the 19 samples with late‐fall LAD designations that also had a genetic assignment, 16% genetically assigned to late‐fall and 84% assigned to fall. While our study was not designed to precisely quantify errors in LAD assignments, these results suggest LAD assignments overestimate the number of late‐fall juveniles. We conclude LAD assignments for late‐fall exhibit a high error rate in our dataset similar to that of winter and spring LAD designations (see above).

## 4 DISCUSSION

CV Chinook salmon were historically one of the most complex populations in the world, but have suffered severe losses of diversity and are vulnerable to sudden population collapse (Carlson & Satterthwaite, 2011; Griffiths et al., 2014; Yoshiyama et al., 1998). Human activities are responsible for the declines in diversity, but deliberate management actions may also protect and promote the diversity that remains (Herbold et al., 2018; Yoshiyama et al., 1998). However, effective management of diversity depends on an accurate understanding and monitoring framework for individual components of diversity, which can be difficult to develop and typically becomes more challenging at finer scales. Genetic approaches hold promise for improving monitoring frameworks (Baerwald et al., 2023; Cordoleani et al., 2020; Meek et al., 2020; Nelson et al., 2022), but the accuracy of genetic approaches can face challenges from low differentiation as well as heterogeneity in sample qualities. Here, we utilized thousands of SNPs from next generation sequencing data and showed that a step‐wise approach based on a hierarchy of genetic differentiation, thorough threshold development, and method validation can address these challenges and facilitate confident assignments to origin even for subpopulations with low levels of differentiation in highly heterogeneous datasets. Thus, our study shows the feasibility of genetics to not only improve monitoring of major populations, but also expand and incorporate monitoring of subpopulation‐level diversity.

Wild spring run Chinook salmon in the CV have experienced severe declines in diversity, having lost approximately 80% of historical subpopulations, and are listed as Threatened under the ESA (US Office of the Federal Register, 1999). Protecting the remaining diversity of spring‐run Chinook is therefore critical. However, a lack of understanding of extant spring‐run diversity and the challenges of monitoring have hindered the development of appropriate management actions (Cordoleani et al., 2020; Nelson et al., 2022). The most apparent remaining components of spring run diversity are the distinct subpopulations within the spring‐run. Mill, Deer, and Butte Creeks represent the largest remaining wild spring‐run subpopulations, and each occupies markedly different tributary habitats (Yoshiyama et al., 1998). Butte Creek spring‐run spawning habitat is in relatively low elevation Northern Sierra geology with historically precipitation‐driven flows (flows are now influenced by human regulation). Mill and Deer Creek spring‐run spawn in high elevation volcanic habitat with substantial groundwater inputs (Cordoleani et al., 2020). Year‐to‐year variations in environmental factors likely play out somewhat differently between these locations, but how each tributary contributes to overall population dynamics in the face of varying environmental conditions is poorly understood (Cordoleani et al., 2020). The ability to identify Butte Creek and Mill/Deer spring‐run Chinook salmon with reasonable confidence in locations and at life‐stages where they would otherwise be indistinguishable provides a powerful tool to improve understanding of subpopulation dynamics and account for both subpopulation groups in monitoring and management.

The strong environmental differences between Mill, Deer, and Butte Creeks are likely to drive phenotypic differences between the spring‐run subpopulations as well. Fish size and occurrence are thought to be important indicators of variation (Miller et al., 2010; Sturrock et al., 2015) and fish success (Duffy & Beauchamp, 2011; Woodson et al., 2013). Diversity in juvenile migration characteristics can be an especially important contributor to population stability, as adult returns are strongly impacted by juvenile survival rates (Sturrock et al., 2015). Migration is a recurring behavior within the animal kingdom in which animals benefit through increased growth or reproductive opportunities by coordinating their movements between spatially segregated resources. Many resources are not static and therefore the timing of migration is an important component of resource acquisition. The match‐mismatch hypothesis suggests that growth and survival are highest when co‐occurring with prey production and this match or mismatch with peak production may in part explain variability in recruitment (Cushing, 1990). Juvenile success depends on alignment of phenotypic characteristics (e.g., growth, timing, habitat choices, etc.) with environmental factors, and previous work using otolith microchemistry has demonstrated that alternative juvenile strategies within individual spring‐run subpopulations can have widely varying success between years (Cordoleani et al., 2021). In altered landscapes, like the California CV and San Francisco Estuary, disconnect between migratory routes and migratory cues can lead to mismatches that become potentially catastrophic (Budy et al., 2002). Understanding the consequences of human modified landscapes and how variation in migration timing may ensure long‐term stability is critical for species persistence and resource management. Here, we identified a significant difference in median juvenile outmigration timing through the delta between spring run subpopulations, with the median date of Mill/Deer Creek juveniles occurring approximately 2 weeks before Butte Creek juveniles in our data (late March vs. mid‐April). In addition, the earliest Mill/Deer fish were detected approximately 2 months before the earliest Butte individual. This early pattern for Mill/Deer spring‐run was notably consistent across years. Given the high variability of environmental conditions during the outmigration period (e.g., from precipitation, changes in snowmelt rate, the timing of ocean upwelling, etc.), the difference in outmigration timing between juveniles from separate subpopulations would result in them experiencing differential environments not only in their natal tributaries, but also during the course of their outmigration through the mainstem river, the delta, and their early ocean residence (García‐Reyes & Largier, 2012). Thus, subpopulations contribute to the portfolio of spring‐run Chinook through exposure to different environmental conditions at every point of the juvenile life stage.

Fall run Chinook salmon in the CV have experienced severe declines in diversity as a result of human‐mediated homogenization and synchronization, and the weak portfolio of the fall‐run is implicated in recent population collapses and fishery closuresCarlson & Satterthwaite, 2011; Williamson & May, 2005; Yoshiyama et al., 1998). The most distinct component of remaining diversity within the fall run ESU is the late‐fall run. While efforts are made to monitor late fall‐run juveniles during their outmigration using LAD criteria, our data suggests this method of assignment has a high error rate (e.g., see Figure 6f), as is also seen in LAD designations for winter and spring runs. Low levels of genetic differentiation present a challenge to incorporating genetic assignments for late‐fall run into monitoring. Here, we show that, with careful validation and threshold development, error rates can be controlled and reduced to levels low enough to allow efficacious assignments. Thus, our results demonstrate that incorporating genetic assignments for late fall‐run into routine monitoring could improve the ability to both study and manage this important component of diversity.

As with the spring run subpopulations, the late‐fall and fall runs are exposed to different environmental conditions. The late‐fall run utilizes spawning habitat at the current upper most limits of anadromy in the CV (most historical late fall‐run habitat has been impounded behind Shasta dam) (Yoshiyama et al., 1998). In contrast, the fall‐run spawns in multiple tributaries throughout the basin, but is strongly homogenized and exhibits a high degree of synchrony despite geographic breadth (Carlson & Satterthwaite, 2011; Williamson & May, 2005; Yoshiyama et al., 1998). This synchrony, as well as the outmigration patterns observed in this paper, are likely strongly influenced by the substantial numbers of hatchery‐origin fish within the fall run. Late‐fall Chinook salmon exhibit phenotypic difference from the fall‐run in both their adult migration timing and their typical age at return (the late‐fall produces a higher percentage of 4‐year old spawners than the fall‐run, which are more often 3‐year‐olds) (Satterthwaite et al., 2017) as well as differences in the timing and duration of juvenile life events (e.g., incubation, emergence, etc.) (USFWS, 1995; Yoshiyama et al., 1998). Here, we find strongly significantly different median migration times through the delta for late fall‐ and fall‐run juveniles. This difference is greater than any other comparisons made in this study. Notably, the earlier outmigration of late‐fall juveniles observed here is consistent with prior non‐genetics based work describing the outmigration patterns of fall and late‐fall run juveniles (e.g., Yoshiyama et al. (1998) reports primary outmigration timing is November through May for late fall‐run and March through July for fall‐run). As with the spring run subpopulations described above, differences in outmigration timing are expected to result in different environmental exposures for late fall‐ and fall‐run juveniles at every point of the juvenile life stage. Thus, the late fall‐run embodies critical diversity within the fall‐run ESU throughout its life cycle, and genetic tools can improve the ability to study and monitor this diversity. Further, migration timing has important implications for survival to adulthood, and peak survival timing varies from year to year (Scheuerell et al., 2009), so the maintenance of diversity in timing may play a key role in survival dynamics. Similarly, Achord et al. (2007) showed diversity of migration times resulted in different salmon populations encountering different prey abundances in the estuary. These results illuminate the importance of maintaining juvenile migration diversity, and synchrony in these populations is likely gained by a combination of attributes.

Every run of CV Chinook salmon has experienced the extirpation of many historical distinct subpopulations. The phenotypic diversity identified in our analyses of the few remaining subpopulations highlights that the loss of these extirpated populations would have been accompanied by the loss of the distinct phenotypic diversity they embodied. It also highlights the potential benefits of restoration actions. For example, the diversity harbored between the 14 spring run subpopulations that have been extirpated was not well characterized prior to their loss, but our results finding distinct juvenile outmigration distributions between the remaining subpopulations suggests each historical subpopulation harbored distinct phenotypic diversity as well. Furthermore, we did not examine diversity within winter‐run subpopulations because only a single, very small population currently exists due to blocked access to all historical spawning habitat. However, four winter‐run subpopulations historically existed (Lindley et al., 2004), and likely exhibited diversity that supported the winter‐run portfolio. Efforts are in development to restore spring‐run Chinook to the San Joaquin basin and restore winter‐run to the upper Sacramento. Our results suggest that if the efforts are successful, the restored subpopulations may evolve local diversity that would strengthen the portfolio of the larger populations.

This study compared the outmigration timing and size distributions of juvenile Chinook from different populations and subpopulations and found highly significant differences for all comparisons. However, while the design of this study is capable of evaluating whether groups of samples were derived from the same or different distributions, it is not necessarily sufficient to describe the individual distributions of each population or subpopulation with high precision (e.g., due to issues with variation in sampling effort, a large influx of fall juveniles potentially masking less‐abundant populations at certain times, etc.). Nevertheless, this work demonstrates the power of genomics‐based population assignments to aid the study and management of intraspecific phenotypic diversity.

Future work incorporating genomics‐based assignments into other powerful approaches such as life‐cycle models and juvenile production estimates (Cordoleani et al., 2020; Nelson et al., 2022) will fill important data gaps and address management challenges. Furthermore, the ability to confidently assign individuals to subpopulations of origin provides a means to account for subpopulation diversity that is critical for maintaining and bolstering the diversity portfolio of CV Chinook salmon.

## CONFLICT OF INTEREST STATEMENT

The authors declare no conflicts of interest.

## Supporting information


Appendix S1.

## Data Availability

Information and data used to complete the analyses described in this manuscript (including sample metadata, single read allele call files, scripts used for analysis, and results files) are publicly available on the Dryad Digital Repository (DOI: 10.5061/dryad.280gb5mxx).
