# Supplementary material for: Genomics and 20 years of sampling reveal phenotypic differences between subpopulations of outmigrating Central Valley Chinook salmon
Source: Evol Appl. 2024 Jun 3;17(6):e13705. doi: 10.1111/eva.13705 (PMC11146144; doi:10.1111/eva.13705)
Supplement: Supplementary file 1 — Appendix S1. [file EVA-17-e13705-s001.pdf]

Supplemental Table 1

Dataset	Origin	Type of data	Population (n)
Training set	Meek et al., (2020)	100 base-pair single-end RAD (*SbfI*)	Winter run (n=30)*
			Spring run—Butte Creek (n=30)*
			Spring run—Mill/Deer Creeks (n=56)*
			Late-fall run (n=30)*
			Fall run** (n=302)*
Validation set	Baerwald et al., (2023)	150 base-pair paired-end whole genome	Winter run (n=6)
			Spring run—Butte Creek (n=7)
			Spring run—Mill/Deer Creeks (n=30)
			Late-fall run (n=6)
			Fall run*** (n=42)
Chipps Island juveniles	Current study	150 base-pair paired-end RAD (*SbfI*)	Unknown (n=5287)****

Supplemental Table 1: Information on sample sets used in this study.
*Number of samples after excluding samples with fewer than 500,000 aligned reads (see Methods).
**See Figure 1 for sampling locations of Fall run.
***Fall run sampling locations include STN, MER, DER, COL, MIL, BUT, TOU (see Figure 1 for map of locations).
****Unknown (n=4632) after excluding samples with fewer than 1,000 aligned reads.

Supplemental Table 2

Assignment Step	Missingness Thesholds to Remove	Posterior Thresholds to Remove
Step 1: Winter vs Spring/Fall/Late-fall	>= 99%	0.3-0.7
Step 2: Wild Spring vs Fall/Late-fall	>= 97%	0.3-0.7
Step 3a: Spring Butte vs Spring Mill/Deer	>=80%	0.3-0.7
Step 3b: Fall vs Late-fall	>=80%	0.45-0.55

Supplemental Table 2. Missingness and posterior thresholds. For a give step, samples with percents of missing data above the missingness threshold or whose assignment posterior probability fell within the posterior threshold range were not given a population assignment for that step and were excluded from all downstream analyses.

Supplemental Figure 1

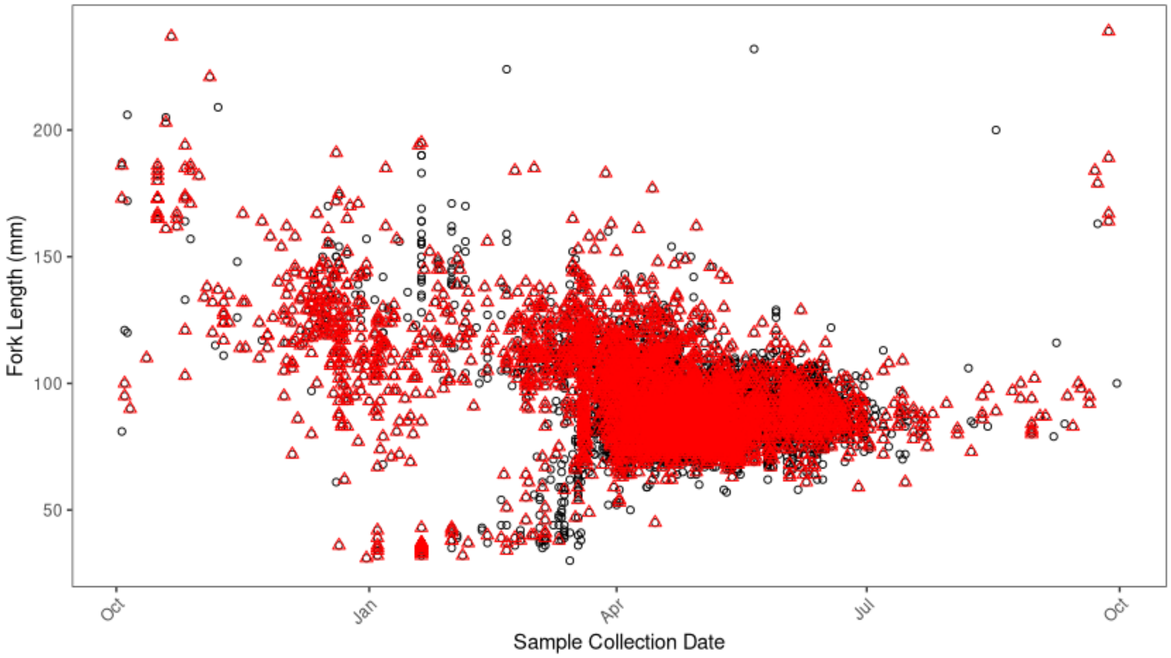

Supplemental Figure 1. Samples used in this study compared to all juvenile samples available in the CDFW tissue archive (collected at Chipps Island). Black circles represent all archive samples. Red triangles are samples selected from the archive for use in this study.

Supplemental Figure 2

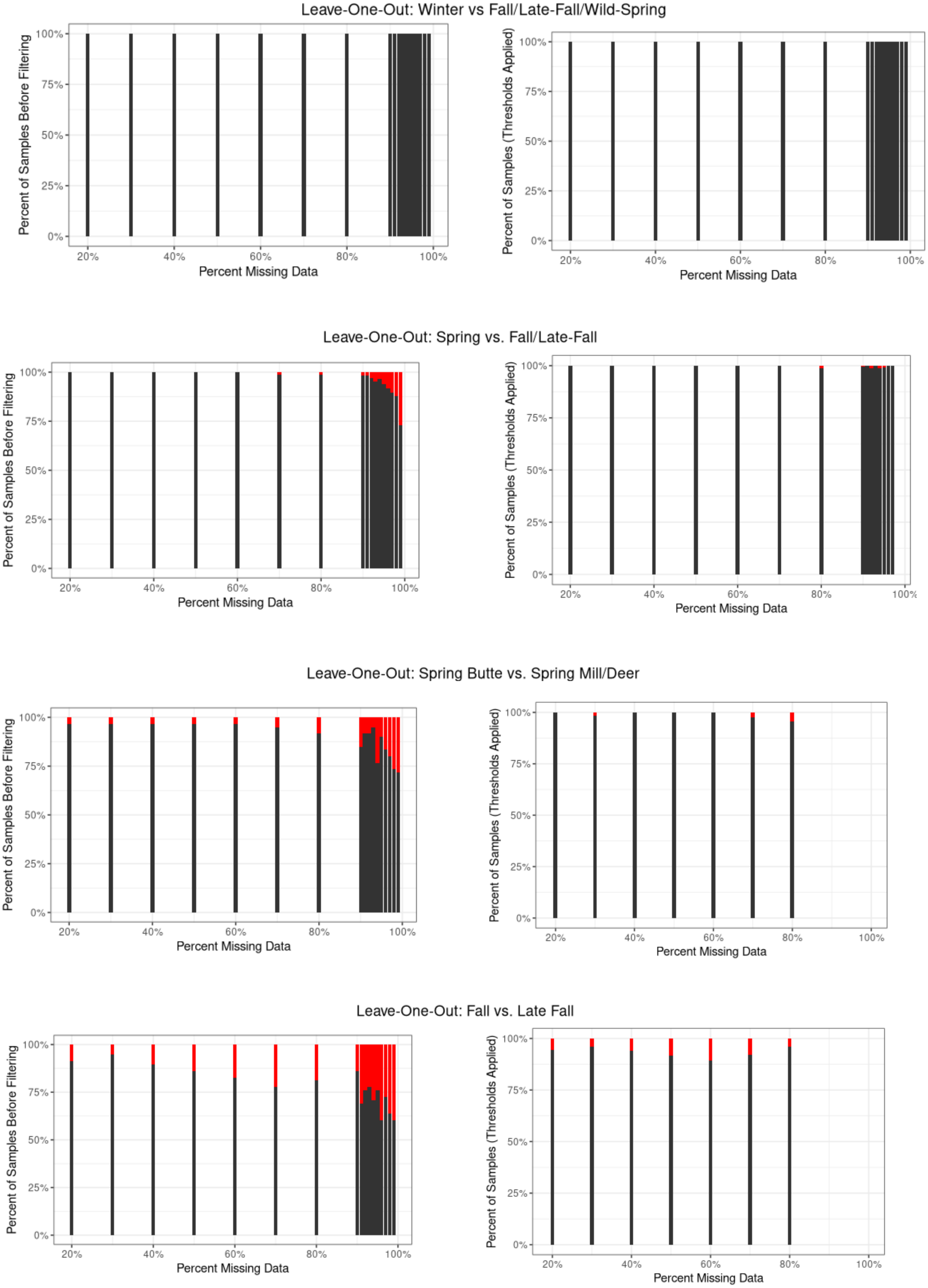

Supplemental Figure 2. Comparison of error rates in leave-one-out analysis before and after posterior and missingness thresholds were applied. Left column contains results prior to applying thresholds. Right column contains results after applying thresholds. Black indicates samples that were assigned to their correct population or subpopulation. Red indicates incorrect assignments.

Supplemental Figure 3

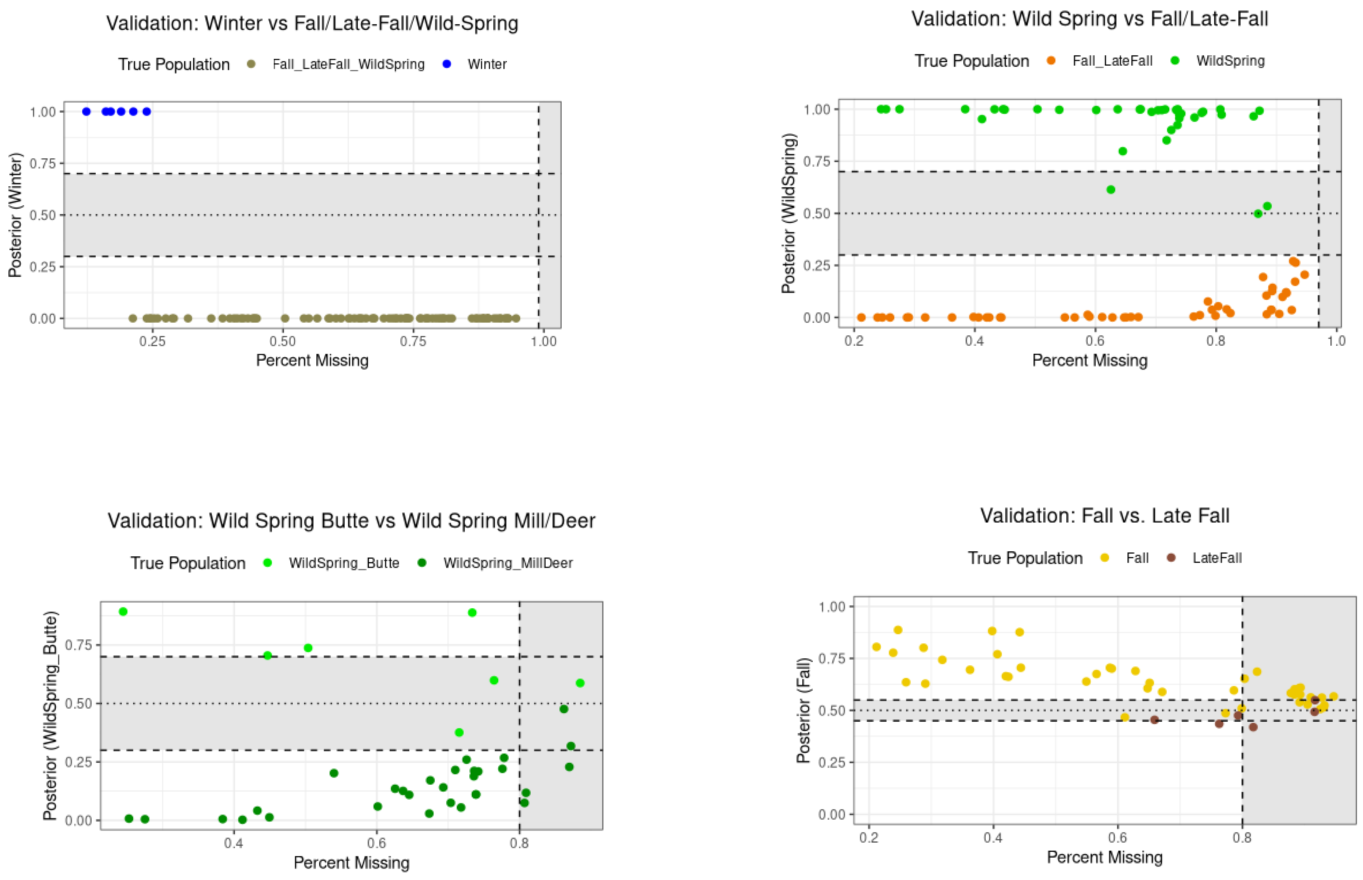

Supplemental Figure 3. Validation sample results. Points represent individual samples from the validation dataset. Color indicates the true population or subpopulation of origin. Gray areas represent posterior and missingness thresholds.

Supplemental Figure 4

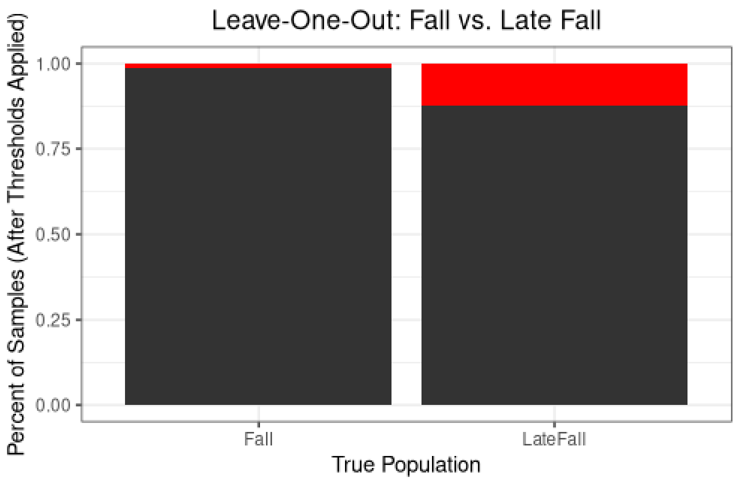

Supplemental Figure 4. Proportion of fall and late-fall samples correctly and incorrectly assigned during leave-one-out analysis after posterior and missingness thresholds were applied. Black indicates correct assignments while red indicates incorrect assignments. This shows that most errors at this step are the result of true late-fall samples being incorrectly assigned as fall. It is rare for true fall to be incorrectly assigned as late-fall.

Supplemental Figure 5

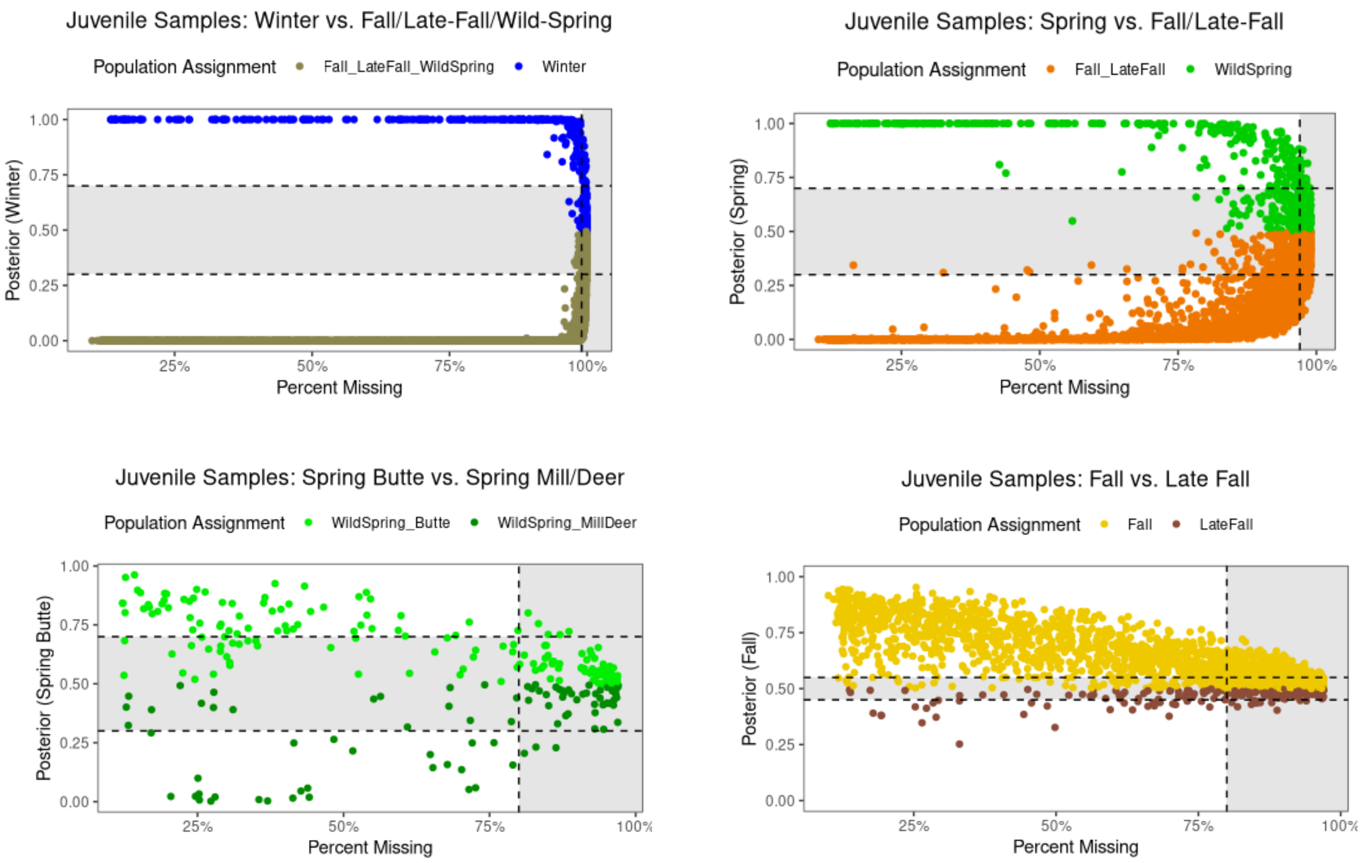

Supplemental Figure 5. Juvenile sample population and subpopulation assignments. Each point represents an individual sample. Gray areas and dashed lines represent posterior and missingness thresholds. Samples that fell within a gray area were not given an assignment and were excluded from all downstream analyses.
